# A New Potential Energy Surface of the PO^+^‑H_2_ Complex and Intermolecular Rovibrational State Calculations

**DOI:** 10.1021/acs.jpca.5c02638

**Published:** 2025-07-02

**Authors:** Hervé Tajouo Tela, Cheikh T. Bop, François Lique, Steve Ndengué

**Affiliations:** † ICTP-East African Institute for Fundamental Research, University of Rwanda, P.O. Box 3900 Kigali, Rwanda; ‡ Physics Department, Khalifa University, P.O. Box 127788 Abu-Dhabi, United Arab Emirates; § Univ. Rennes, CNRS, IPR (Institut de Physique de Rennes) − UMR 6251, F-35000 Rennes, France; ∥ Department of Physics and Astronomy, 3776 Haverford College, Haverford, Pennsylvania 19041, United States

## Abstract

The recent detection of the phosphorus monoxide cation (PO^+^) in the interstellar medium (ISM) has generated considerable interest in its collisional excitation and reactivity in such environments. Due to the difficulties in conducting laboratory experiments in these extreme environments, theoretical calculations have become essential to model the excitation and reactivity of PO^+^. In this context, several theoretical studies have been conducted to better understand its abundance and impact on interstellar chemical processes. An important aspect of these studies is the accurate characterization of their electronic interaction with the surrounding gas constituents. We present here a new four-dimensional potential energy surface (PES) for the interaction between the PO^+^ cation and the H_2_ molecule, the dominant species in the cold ISM, using the explicitly correlated coupled cluster method with single, double, and perturbative triple excitations [CCSD­(T)-F12**a**]. The rigid rotor PES provides a global representation of the PO^+^-H_2_ interaction, and presents a unique global minimum with a well depth of 1252.88 cm^–1^. We subsequently characterized the rovibrational states of the PO^+^-H_2_ complex, up to a total angular momentum *J* of 3, by solving the nuclear Schrödinger equation with the block-induced relaxation procedure implemented in the Heidelberg Multi-Configuration Time Dependent Hartree (MCTDH) package. We obtained zero-point energies of 422.201 cm^–1^ for PO^+^-*para*-H_2_ and 487.805 cm^–1^ for the PO^+^-*ortho*-H_2_ complex. This corresponds to dissociation energies (*D*_0_) of 830.679 and 765.075 cm^–1^for PO^+^-*para*-H_2_ and of 487.805 cm^–1^ for the PO^+^-*ortho*-H_2_ complex. We hope that the present theoretical results will stimulate experimental studies of the PO^+^-H_2_ complex in order to validate the predictions reported in this work.

## Graphical abstract

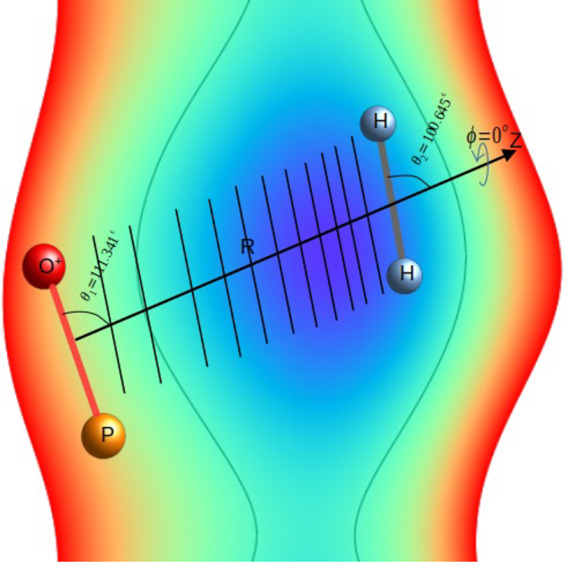

## Introduction

Phosphorus (P) is a fundamental element of life, and the chemistry of phosphorus in the interstellar medium (ISM) has become increasingly important in astrobiology.^1^ Species containing P element, such as PO and PH_3_, have been detected in space and are believed to contribute to the formation of complex interstellar biogenic molecules.^2^

Recently, the PO^+^ ion was detected by Rivilla et al.^3^ in the *G* + 0.693 – 0027 molecular cloud located in the SgrB2 region of the center of the Galaxy. Its estimated abundance was found to be 4.5 × 10^–12^ relative to molecular hydrogen.^3^ The formation of the PO^+^ ion in such media occurs through the reaction of P^+^ with OH or O_2_.^3^ The ionization of PO is also a possible path for the formation of PO^+^.^3^ Despite a low fractional abundance, PO^+^, together with P^+^, has a predominant role in the chemical network of P.

Since its detection, the rotational energy transfer of PO^+^ induced by molecular hydrogen collisions^4,5^ has become a focal point for studies. Indeed, H_2_ is, by far, the most abundant molecule in molecular clouds and is mainly responsible for the PO^+^ excitation in such media. Such studies are especially important for astronomers seeking to estimate the abundance and emission of PO^+^ in the interstellar medium (ISM) using non-Local Thermodynamic Equilibrium (LTE) radiative transfer models.^6^

The study of the collisional excitation of PO^+^ by H_2_ requires prior determination of the interaction of PO^+^ with H_2_. As such, the interaction of the PO^+^ ion with H_2_ was modeled in recent work by Tonolo et al.^4^ and Chahal et al.^5^ The PO^+^-H_2_ potential energy surface (PES) of Tonolo et al.^4^ was calculated using the explicitly correlated coupled cluster method with single, double, and perturbative triple excitations [CCSD­(T)-F12**a**] method^7–9^ in conjunction with the augmented correlation consistent quadruple-ζ basis set augmented by an additional *d*-function for P, the aug-cc-pV­(Q + *d*)­Z (hereafter AV­(Q + *d*)­Z) basis set.^10^ The PO^+^-H_2_ PES of Chahalet al.^5^ was calculated using a coupled-cluster method together with an extrapolation to the complete basis set (CBS) limit. Both PESs are considered rigid monomers.

In both situations, PES was used to study the inelastic scattering of PO^+^ by H_2_. Although these PESs were computed at a similar level of accuracy and looked globally similar, the scattering results turned out to be significantly different. In fact, the collisional data that resulted from the two PESs differed by more than a factor of 2 for most of the transitions.^5^ Such a deviation is quite surprising and cannot be easily explained.

In light of these differences, the calculation of new collisional data based on a new PO^+^-H_2_ PES at the highest theoretical level possible seems essential to resolving this discrepancy. This work represents the first step in this process: computing a new PO^+^-H_2_ PES for the system and comparing it to the two previously developed PESs. In addition, we present calculations of the rovibrational levels of the PO^+^-H_2_ complex, which could serve as a reference for future experimental investigations to validate the precision of the new PES. To our knowledge, no such calculations have been reported despite the availability of recent PESs that describe the PO^+^-H_2_ interaction.

This work is organized as follows. In the next section, we describe the methodology and computational procedure followed for the PES calculation and quantum dynamical simulations using the MCTDH package. We then present and discuss our results, and after summarizing our work, we mention future avenues of research for this system and others.

## Methods

### Potential Energy Surface

#### Potential Energy Surface and Its Analytical Representation

The interaction potential energy between PO^+^(^1^Σ^+^) and H_2_(^1^Σ_*g*_^+^) is calculated using the rigid-rotor approximation. The H_2_ bond length *r*_H_2__ is the internuclear distance averaged over the ground vibrational wave function (*r*_H_2__ = 0.767 Å). The PO^+^ bond length *r*_PO^+^_ is fixed at the distance corresponding to the equilibrium geometry of PO^+^ (*r*_PO^+^_ = 1.42499 Å),^11,12^ as to the best of our knowledge, the ground vibrational wave functions are not available in the literature. The use of vibrationally averaged (*r*_0_) instead of equilibrium geometries (*r*_e_) is recommended in PES calculations as cross sections resulting from such interaction potentials agree better with those computed in a full-dimensional PES including vibrational effects. However, Faure et al.^13^ showed that rigid-rotor PESs, based on averaged as well as equilibrium geometries, yield cross sections that agree reasonably well with experimental measurements. Furthermore, *r*_0_(PO^+^) is not expected to differ strongly from *r*_e_(PO^+^). Therefore, using the PO^+^ equilibrium geometry instead of its vibrationally averaged internuclear distance likely has minor effects on the accuracy of the PES, and both molecules can be considered in their ground vibrational states. The PO^+^-H_2_ four-dimensional PES is expressed as a function of Jacobi coordinates as defined in Figure 1. Here, *R* denotes the distance between the centers of mass of the two monomers, the angles θ_1_ and θ_2_ describe the orientation of PO^+^ and H_2_, respectively, with the colliding axis (*Z*), and ϕ represents the dihedral angle between the half-planes containing PO^+^ and H_2_.

**Figure 1:**
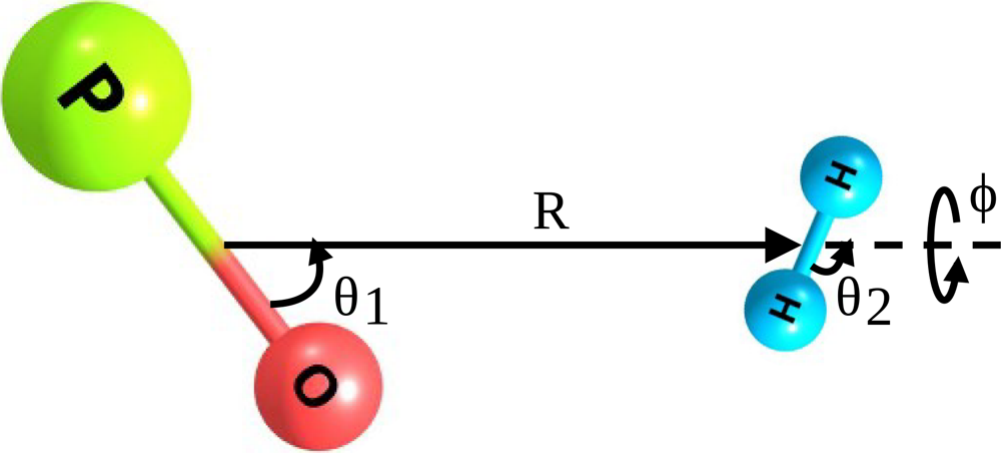
Definition of the PO^+^-H_2_ Jacobi coordinate system used to compute the PES.

For all electronic structure calculations, we use the CCSD­(T)-F12a (hereafter denoted CCSD­(T)-F12) in conjunction with the augmented correlation consistent triple-ζ (AVTZ) Gaussian basis set, as implemented in version 2015 of the MOLPRO quantum chemistry package.^10,14,15^ For explicit correlation calculations, we used the VTZ/JKFIT and AVTZ/MP2FIT complementary basis sets of Weigen for the evaluations of the density fitting (DF) and resolution identity (RI).^16^ In practice, core–valence effects were estimated, including all electrons except those in 1s of oxygen and 1s, 2s, and 2p of phosphorus. The contribution of the 2p orbital of phosphorus in the interaction energy, estimated at *R* = 5.25 *a*_0_, ϕ = 0°, θ_2_ = [80 – 120]°, and θ_1_ = 112°, turns out to be less than 3 cm^–1^. This level of theory, CCSD­(T)-F12/AVTZ, is known to be very high and has been used in the literature to calculate PESs and derive bound states, differential cross sections, and integral cross sections that agree fairly well with experimental measurements.^17–20^ The potential energy surface is constructed with 53 *R*-grid points (ranging from 4 to 20 *a*_0_) and a 15-point Gauss-Legendre quadrature for θ_1_. In addition, a 9-point Gauss-Chebyshev scheme (for ϕ) and a 5-point Gauss-Legendre scheme (for θ_2_) are used in the calculations to characterize the rotational motion of H_2_. The size consistency error, arising from the evaluation of the perturbative triple excitations,^8^ is corrected for all geometries by subtracting from all energies the potential obtained at *R* = 200 *a*_0_

$$V\left(R,\alpha \right)=V\left(R,\alpha \right)-V\left(R=200a_{0},\alpha \right)$$ 1

where α stands for {θ_1_, θ_2_, ϕ}. To consider the ionic nature of the collisional system, we computed additional interaction energies from *R* = 20 *a*_0_ to *R* = 50 *a*_0_ using the standard CCSD­(T) method,^21^ as CCSD­(T)-F12 may not consistently capture the long-range interaction. The potential energies obtained with the two levels of theory exhibit differences of less than 3% at *R* = 20 *a*_0_. Consequently, the two data sets [*V* (*R* ≤ 20 *a*_0_, α) and *V* (*R* ≥ 21 *a*_0_, α)] are seamlessly connected by using a cubic spline routine. For all ab initio points, the errors resulting from basis set superposition are removed using the counterpoise method.^22^

$$V\left(R,\alpha \right)=E_{{\mathrm{PO}}^{+}-H_{2}}^{H_{2}\mathrm{PO}}\left(R,\alpha \right)-E_{{\mathrm{PO}}^{+}}^{H_{2}\mathrm{PO}}\left(R,\alpha \right)-E_{H_{2}}^{H_{2}\mathrm{PO}}\left(R,\alpha \right)$$ 2

Here, *E*_A_^B^ stands for the energy of monomer A computed considering the atomic orbitals of all atoms in complex B. Based on different geometries, *R* = 5.25 *a*_0_, ϕ = 0°, θ_2_ = [80 – 120]°, and θ_1_ = 112°, these errors were estimated to be about 15 cm^–1^.

To obtain an analytical representation of the PO^+^-H_2_ ab initio PES, we expand the interaction potential over contracted normalized bispherical harmonics as follows:

$$V\left(R,\theta_{1},\theta_{2},\phi \right)=\underset{L_{1}L_{2}L}{\sum }v_{L_{1}L_{2}L}\left(R\right)A_{L_{1}L_{2}L}\left(\theta_{1},\theta_{2},\phi \right)$$ 3

eq 4 introduces the definition of bispherical harmonics,

$$A_{L_{1}L_{2}L}\left(\theta_{1},\theta_{2},\phi \right)=\overset{\min\left(L_{1},L_{2}\right)}{\underset{M=0}{\sum }}\beta_{i}\frac{\alpha i}{\left(1+\delta_{M0}\right)}\left(\begin{array}{lll} L_{1} & L_{2} & L\\ M & -M & 0 \end{array}\right)\times P_{L_{1}M}\left(\theta_{1}\right)\times P_{L_{2}M}\left(\theta_{2}\right)\cos\left(M\phi \right)$$ 4

with

$$\beta_{i}=2{\left(-1\right)}^{M}\left(\frac{2L+1}{2\pi }\right)\frac{1}{\sqrt{4\pi }}{\left(-1\right)}^{\left(L_{1}+L_{2}\right)}$$ 5

and

$$\alpha_{i}=\frac{\sqrt{\left(2L_{1}+1\right)\left(L_{2}+1\right)}}{2}\sqrt{\frac{\left(L_{1}-M\right)!\left(L_{2}-M\right)!}{\left(L_{1}+M\right)!\left(L_{2}+M\right)!}}$$ 6

Here, *L*_1_ and *L*_2_ are associated with the rotational motion of PO^+^ and H_2_, respectively. They take integer values, starting from 0, up to *L*_1max_ = 14 and *L*_2max_ = 4 for *L*_1_ and *L*_2_, respectively. By definition, *L* = |*L*_1_ – *L*_2_|, ···, *L*_1_ + *L*_2_ and *L*_2_ is multiple of 2 due to the homonuclearity of H_2_, with the additional constraint that (*L*_1_ + *L*_2_ + *L*) is even. This resulted in a total of 122 expansion terms *v*_*L*_1_ *L*_2_ *L*_(*R*) to represent the PES. The relative mean deviation generated by the analytical fit (eq 3) is 1.4% at *R* = 4.75 *a*_0_, and it remains below 1% at all other distances. The overall RMSE of the analytical fit is 1.98 cm^–1^ for all points with *R* between 4 and 50 *a*_0_ and goes up to 2.63 cm^–1^ for *R* between 4 and 20 *a*_0_. The relative error is 0.14 and 0.18%. That is the analytical representation that not only describes faithfully the ab initio data in the well, but also in the long-range.

### Rovibrational State Calculations with MCTDH

#### MCTDH Method and the Kinetic Energy Operator

The rovibrational spectrum of the PO^+^-H_2_ complex is studied with the MCTDH method.^23–26^ MCTDH is a time-dependent method in which each degree of freedom (DOF) is associated with a small number of orbitals or single-particle functions (SPFs), which, through their time dependence, allow an efficient description of the quantum dynamical process. The MCTDH wave function is expanded as a weighted sum of time-dependent Hartree products:

$$\Psi (Q_{1},...,Q_{f},t)=\sum_{j_{1}=1}^{n_{1}}\cdot \cdot \cdot \sum_{j_{f}=1}^{n_{f}}A_{j_{1}\cdot \cdot \cdot j_{f}}(t)\prod_{\kappa =1}^{f}\phi_{j_{\kappa }}^{\left(\kappa \right)}(Q_{\kappa },t)=\sum_{\Lambda }A_{\Lambda }\Phi_{\Lambda }=\sum_{j=1}^{n_{k}}\varphi_{j}^{\left(k\right)}\Psi_{j}^{\left(k\right)}$$ 7

where *f* is the number of DOF of the system, *Q*_1_, ···, *Q*_*f*_ are the nuclear coordinates, *A*_Λ_ ≡ *A*_*j*_1_···*j*_*f*__ are the MCTDH expansion coefficients, and ϕ_*j*_κ__^(κ)^(*Q*_κ_, *t*) are the *n*_κ_ SPFs associated with each degree of freedom κ (i.e., they form a time-dependent variable basis along κ).

The subsequent equations of motion for the coefficients and SPFs are derived after substituting the wave function *ansatz* into the time-dependent Schrödinger equation. To solve the equations of motion, the κ SPFs are represented on a (fixed) primitive basis, here a discrete variable representation (DVR) grid^27–29^ of *N*_κ_ points:

$$\varphi_{j_{\kappa }}^{\left(\kappa \right)}\left(Q_{\kappa },t\right)=\overset{N_{\kappa }}{\underset{i_{\kappa }=1}{\sum }}c_{i_{\kappa }j_{\kappa }}^{\left(\kappa \right)}\left(t\right)\chi_{i_{\kappa }}^{\left(\kappa \right)}\left(Q_{\kappa }\right)$$ 8

where ideally the *n*_κ_ in eq 7 is such that *n*_κ_ ≪ *N*_κ_.

We use here, as we did in previous work on van der Waals systems,^30,31^ the block-improved relaxation^32–36^ method available in the Heidelberg MCTDH package, to calculate the rovibrational states of the system. This method has been described in detail before.^24,26,37^ MCTDH calculations are the most efficient when the Hamiltonian is expressed as a sum of products (SOP), which is a weighted sum of the product of functions expressed in each mode (or a combination of modes). The Kinetic Energy Operator (KEO) is usually expressed in the required form when polyspherical coordinates, such as the Jacobi coordinates, are used in this study. Here, we do not work in the Body-Fixed (BF) frame but instead use the *E*_2_ frame, as was done in previous work.^38–40^ The parametrization of the Jacobi coordinates in the *E*_2_ frame^31,40–42^ is shown in Figure 2. The *E*_2_ frame^41^ is defined with its *z*-axis aligned parallel to *R*→, the vector connecting the centers of mass of the two molecules. The two angles (θ_1_, ϕ_1_) determine the orientation of the PO^+^ molecule in our *E*_2_ frame, while the other two spherical angles (θ_2_ and ϕ_2_) define the orientation of the H_2_ molecule.

**Figure 2:**
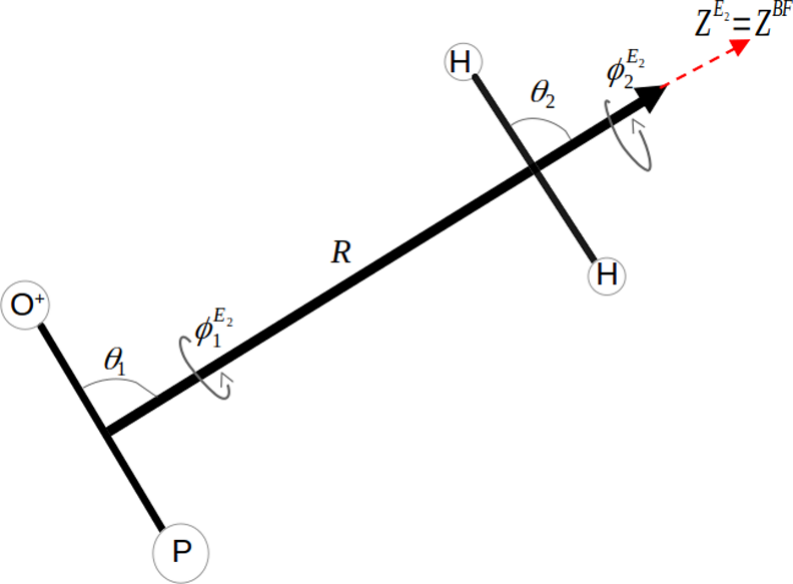
Jacobi *E*_2_ spherical coordinate system for the PO^+^-H_2_ complex: polar and azimuthal angles (θ_1_, ϕ_1_^*E*_2_^) and (θ_2_, ϕ_2_^*E*_2_^) defining PO^+^ and H_2_ orientations relative to the *E*_2_ plane.

#### Potential Energy Surface Representation

The PES computed in this work was originally expressed as eq 3, which is already in the appropriate sum-of-product format for MCTDH calculations. As the calculations are run in the *E*_2_ frame, the angle ϕ is decoupled in that frame as ϕ = ϕ_1_ – ϕ_2_. Here, ϕ_1_ and ϕ_2_ are the out-of-plane torsional angles, defining the rotation of the monomers around the *R* axis. This decoupling does not break the SOP representation but simply adds more terms to the potential representation in its MCTDH implementation.

#### Computational Procedure

The rovibrational state calculations were performed with the MCTDH block-improved relaxation method with a block of 4 states for each calculation, starting from the lowest states and progressively generating more excited states. Table 1 provides a summary of the primitive basis, its range, and the number of single-particle functions (SPFs) used in the calculations of the rovibrational states. On a 32-processor Linux cluster, the calculations ranged from 7 h for the lowest block of 4 states to about 24 h for highly excited states. These time estimates are reasonable for this type of van der Waals system, where we previously noticed that the stabilization of the time-dependent SPFs is rather slow because of the extended landscape of the wells or even their multiplicity.

**Table 1:** Parameters of the Primitive Basis Used for the Rovibrational Calculations of PO^+^-H_2_ ^a^

coordinate	primitive basis	number of points	range	size of SPF basis
*R*	FFT	96	4.0–12.0	7–10
θ_1_	*KLeg*	24	0−π	10–60
ϕ_1_	*K*	15	–7,7	
θ_2_	*KLeg*	9	0−π	10–40
ϕ_2_	*K*	11	–5,5	

a FFT stands for Fast Fourier Transform. *KLeg* is an extended Legendre DVR. *K* stands for the momentum representation of the azimuthal angles ϕ_1_ and ϕ_2_. The spherical angles θ_1_ and ϕ_1_ are for PO^+^, and θ_2_ and ϕ_2_ are for H_2_, both monomers in the rigid rotor approximation. The units for distances and angles are bohrs and radians, respectively.

In this work, we used the masses: 1.00784 u for H, 15.99491461956 u for O, 30.973762 u for P, and 0.00055 u for the electron and the ground state rotational constants^12,43^ *B*_PO^+^_ = 0.784343 cm^–1^ and *B*_H_2__ = 59.322 cm^–1^.^44^ The selection of the primitive basis set was determined through an iterative process. Various combinations of DOF numbers were tested in order to choose the most suitable primitive basis for our calculations. The ones reported in Table 1 yield a convergence of the results to better than 0.02 cm^–1^ for the low-lying states.

Additionally, the number of SPFs for each mode was increased in the calculations from a relatively small value for the lower levels to larger values for the excited states. This value grows rapidly because of the deep well of potential and the rapidly growing density of states with increasing energy. Hence, the primitive basis mentioned in Table 1 is the final configuration (largest values), and all the results presented in this study were derived from these basis parameters. FFT (Fast Fourier Transform): Applied for translational and periodic angular coordinates. *KLeg* (Legendre Polynomials in DVR form): Used for angular coordinates with defined boundaries, providing better localization. *K* (associated Legendre functions): Used in rotational coordinates with coupled angular momentum components. To ensure consistency, we performed a final variation of the single particle function (SPF) basis to achieve complete convergence in the calculations. The different ranges for the single-particle functions (SPFs) associated with ϕ_1_ and ϕ_2_ in Table 1 reflect the different dynamic roles of these angular coordinates in the system. Specifically, ϕ_1_ is associated with the rotation of the heavier PO^+^ fragment, while ϕ_2_ corresponds to the rotation of the lighter H_2_ molecule. As correctly noted by the referee, due to its smaller moment of inertia, H_2_ rotates more rapidly and typically requires a smaller number of primitive basis functions to accurately describe its rotational motion compared to the heavier PO^+^. As shown in Tables 2 and 3, convergence was achieved by increasing the size of the primitive and the SPF basis. As we discuss below, the energy *E*_0_ represents a physical state in Table 2, while the other *E*_1_ to *E*_4_ correspond to nonphysical states (see below) that arise during the calculations.

**Table 2:** Convergence of the Ground State Rovibrational Energy (cm^–1^) of PO^+^-*para*-H_2_ for *J* = 0^a^

SPF	energy				
	*E*_0_	*E*_1_	*E*_2_	*E*_3_	*E*_4_
10/30/20	–830.4600	*–830.4132*	*–830.3611*	*–830.2433*	*–830.2321*
10/40/20	–830.6792	*–830.6128*	*–830.6128*	*–830.4200*	*–830.4600*
10/50/20	–830.6792	*–830.6128*	*–830.6128*	*–830.4200*	*–830.4600*
10/60/20	–830.6792	*–830.6128*	*–830.6128*	*–830.4200*	*–830.4600*
7/60/20	–830.6792	*–830.6128*	*–830.6128*	*–830.4200*	*–830.4600*
10/30/30	–830.4600	*–830.4132*	*–830.3610*	*–830.2431*	*–830.2321*
10/40/30	–830.6792	*–830.6128*	*–830.6128*	*–830.4200*	*–830.4600*
10/50/30	–830.6792	*–830.6128*	*–830.6128*	*–830.4200*	*–830.4600*
7/60/30	–830.6792	*-830.6128*	*-830.6128*	*–830.4200*	*–830.4600*
10/40/40	–830.6792	*-830.6128*	*-830.6128*	*–830.4200*	*–830.4600*
10/50/40	–830.6792	*-830.6128*	*-830.6128*	*–830.4200*	*–830.4600*
7/60/40	–830.6792	*–830.6128*	*–830.6128*	*–830.4200*	*–830.4600*

a In the table, the first column represents the SPF basis, where *a*_1_/*a*_2_/*a*_3_ stands for the number of SPF along the first mode *R*, the second combined mode *KLeg*/*K*, and the third mode *KLeg*/*K*, as suggested in Table 1. States represented in *italic* are fictitious (nonphysical) states.

**Table 3:** Same as Table 2 for PO^+^-*ortho-*H_2_

SPF	energy				
	*E*_0_	*E*_1_	*E*_2_	*E*_3_	*E*_4_
10/30/20	*–768.9351*	*–768.9311*	*–768.0751*	*–768.0751*	*–767.6843*
10/40/20	*–769.1010*	*–769.1010*	*–768.2520*	*–768.2521*	*–767.8155*
10/50/20	*–769.1010*	*–769.1010*	*–768.2520*	*–768.2520*	*–767.8152*
10/60/20	*–769.1010*	*–769.1010*	*–768.2520*	*–768.2520*	*–767.8152*
7/60/20	*–769.1010*	*–769.1010*	*–768.2520*	*–768.2520*	*–767.8152*
10/30/30	*–768.9351*	*–768.9351*	*–768.0751*	*–768.0751*	*–767.6843*
10/40/30	*–769.1010*	*–769.1010*	*–768.2520*	*–768.2520*	*–767.8152*
10/50/30	*–769.1010*	*–769.1010*	*–768.2520*	*–768.2520*	*–767.8152*
7/60/30	*–769.1010*	*–769.1010*	*–768.2520*	*–768.2520*	*–767.8152*
10/40/40	*–769.1010*	*–769.1010*	*–768.2520*	*–768.2520*	*–767.8152*
10/50/40	*–769.1010*	*–769.1010*	*–768.2520*	*–768.2520*	*–767.8152*
7/60/40	*–769.1010*	*–769.1010*	*–768.2520*	*–768.2520*	*–767.8152*

#### Symmetry and Assignment of States

The computational procedure described above, just like in previous work^30,31^ using the MCTDH algorithm in the *E*_2_ frame, helps generate a large number of states, some physical and some unphysical. While calculations performed on slightly similar van der Waals systems (H_2_O-HCN^45^ or H_2_O–H_2_ ^46,47^ ) by other groups used a primitive basis’ constraint such that *K*, the projection of the total angular momentum, satisfies *K* = *m*_*A*_ + *m*_*B*_ with *m*_*A*_ and *m*_*B*_ being the projections of the angular momenta of PO^+^ and H_2_, respectively. The calculations with the MCTDH package do not allow for such flexibility. However, using a procedure we presented in our previous work,^30,31^ we can assign and distinguish the rovibrational states from a wave function analysis. First, as we did in our H_2_O–H_2_’s work, the Σ, Π, and ··· characters of the wave function can be extracted from the MCTDH calculation by looking at the output file of a single-state calculation. Then, summing the average values of the ϕ_1_ and ϕ_2_ DOFs (which correspond to the *m*_*A*_ and *m*_*B*_ used by Wang and Carrington^47^), we can determine *K* as *K* = ⟨ϕ_1_⟩ + ⟨ϕ_2_⟩. This approach not only makes it possible to determine *K* but also filters out physical states from fictitious ones, which are states for which ⟨ϕ_1_⟩ + ⟨ϕ_2_⟩ = *K* > *J*.

The calculations are performed separately for the *para*-H_2_ and *ortho*-H_2_ nuclear spin isomers of H_2_, as in spectroscopic studies (as well as in scattering studies), they can be considered as two distinct species since they are not radiatively (or collisionally) connected. In *ortho*-H_2_, the spin wave function is symmetric with respect to the exchange of nuclei, while in *para*-H_2_, it is antisymmetric. Since protons are Fermions, the total nuclear wave function must be antisymmetric. Consequently, the rotational wave function must be symmetric for *ortho*-H_2_ and antisymmetric for *para*-H_2_. This restriction imposes that the allowed rotational quantum numbers *j* are odd for *ortho*-H_2_ and even for *para*-H_2_.

In Table 2, we show the lowest 5 states of a block-improved relaxation calculation for PO^+^-*para*-H_2_, where only the first level of the 5 calculated is physical. In Table 3, we show the convergence tests for the PO^+^-*ortho*-H_2_ complex: The first physical state appears with an energy higher than the 5 presented and is therefore not displayed in Table 2.

### Rovibrational State Calculations with the Coupled Channel Approach

To validate the MCTDH results, we also calculated the bound states for the total angular momenta *J* = 0 and 1 using the coupled channel approach implemented in the BOUND program.^48^ To perform these calculations, we used the full spherical harmonic expansion of PO^+^-H_2_ PES, with 122 functions. The coupled equations were solved using the modified diabatic log-derivative method.^49^ These 4D calculations were performed for both *para*- and *ortho*-H_2_, and both molecules were taken as rigid rotors with rotational constants *B*_0_ = 59.322 cm^–1^ for H_2_ and *B*_0_ = 0.784343 cm^–1^ for PO^+^. A total of 26 rotational states (i.e., up to *j*_1_ = 25) were included in the PO^+^ basis set while the three lowest rotational states of *para*-H_2_ (*j*_2_ = 0, 2, 4) and *ortho*-H_2_ (*j*_2_ = 1, 3, 5) were considered. The calculations were performed with a propagator step size of 0.01 bohr, and the other propagation parameters were taken as the default BOUND values, where the zero-point energies were found to be 423.901 and 491.497 cm^–1^ for PO^+^-*para*-H_2_ and PO^+^-*ortho*-H_2_, respectively.

## Results and Discussion

### PO^+^-H_2_ Interaction Potential

The global minimum of the PO^+^-H_2_ potential energy surface is located at an intermolecular separation of *R* = 5.181 bohr, with angular configurations defined by θ_1_ = 111.341°, θ_2_ = 100.645°, and dihedral angle ϕ = 0°. Figure 3 displays selected 2D cuts of the PES. In Figure 3a, the plot shows the anisotropy of the interaction with the orientation of H_2_ fixed at θ_2_ = 100.645° and ϕ = 0°. Figure 3b illustrates the impact of H_2_ rotation on the interaction with the PO^+^ geometry fixed at θ_1_ = 111.341° and ϕ = 0°. Figure 3c highlights the anisotropy of the interaction potential with the rotations of both molecules, emphasizing the anisotropy with respect to θ_1_ and θ_2_. Figure 3d presents the 2D contour plots of the PO^+^ - H_2_ PES, with the intermolecular distance fixed at *R* = 5.181 bohr and the angle θ_2_ = 100.645° held constant.

**Figure 3:**
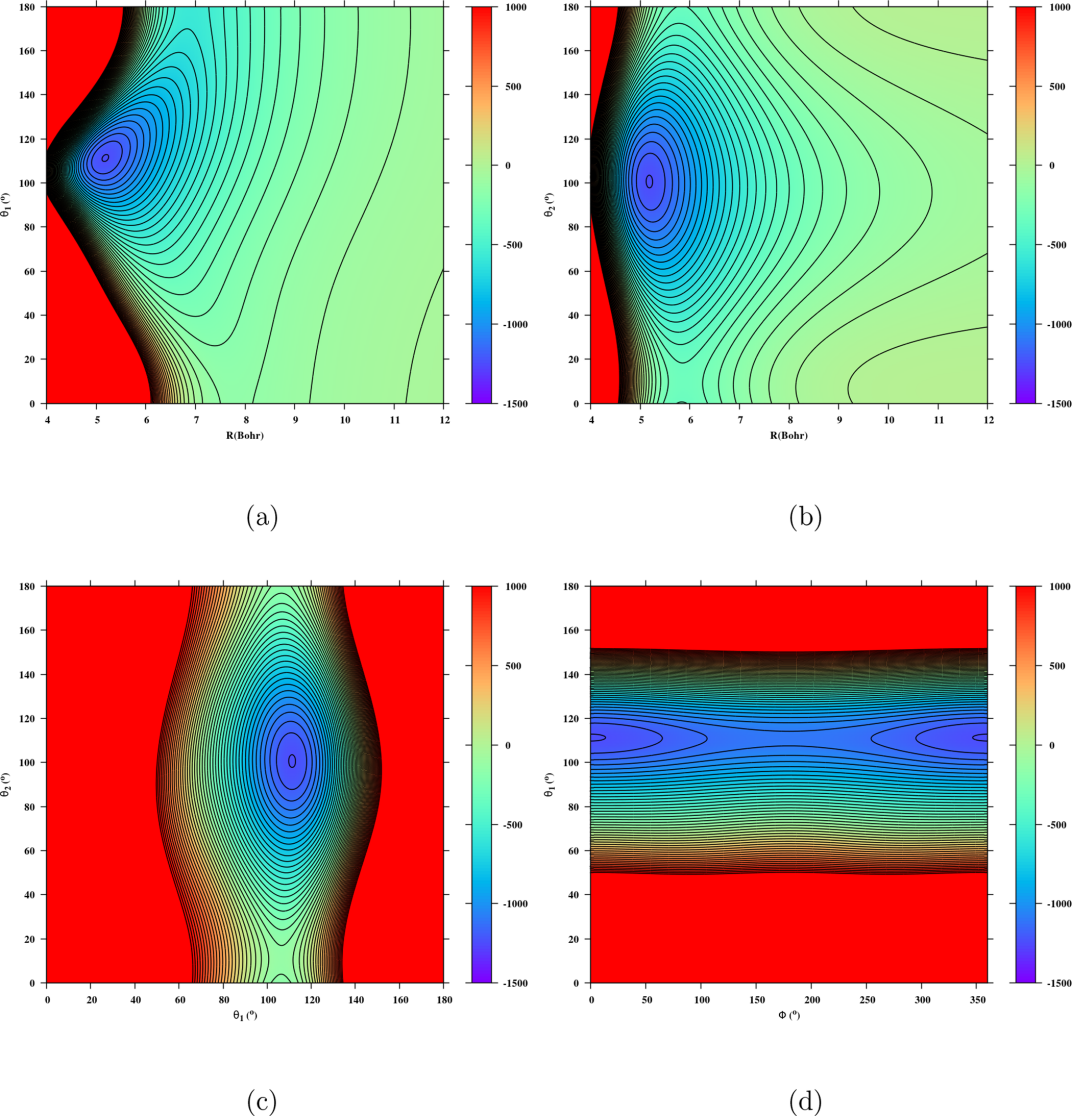
Contour plot of the 2D cut of the 4D PES of PO^+^-H_2_ for fixed θ_2_ = 100.645° and ϕ = 0° (a); contour plot of the 2D cut of the 4D PES for fixed θ_1_ = 111.341° and ϕ = 0° (b); contour plot of the 2D cut of the 4D PES for fixed *R* = 5.181 borh and ϕ = 0° (c) contour plot of the 2D cut of the 4D PES for fixed *R* = 5.181 bohr and θ_2_ = 100.645° (d). The figures show the global minimum *D*_e_ = 1252.88 cm^–1^.

These features show that 4D PES has strong anisotropies with respect to θ_1_ and θ_2_. Table 4 compares the equilibrium positions and well depths of the present PES with those of Tonolo et al.^4^ and Chahal et al.^5^ The equilibrium geometry of the PO^+^-H_2_ complex obtained in this work is in good agreement with previous studies. Minor differences may be due to variations in the computational methods or basis sets. In particular, the dissociation energy (*D*_e_) found in this work (1252.88 cm^–1^) is higher than the one reported by Chahal et al. (1230.18 cm^–1^), while the value derived from the analytical PES of Tonolo et al. (1288 cm^–1^) is higher than the new one. These small differences may suggest a marginally stronger interaction in some calculations, possibly due to improved correlation treatment or a more refined potential energy surface, coming from a higher density of data fitted.

**Table 4:** Equilibrium Position of the PO^+^-H_2_

	*R* (*a*_0_)	θ_1_ (°)	θ_2_ (°)	ϕ (°)	*D*_e_ (cm^–1^)
this work	5.18	111.341	100.645	0	1252.88
Tonolo et al.^4^	5.29	112.291	100	0	1288
Chahal et al.^5^	5.18	110	100	0	1230.18

The left panel of Figure 4 compares the analytical representation of the newly computed 4D PES with that of the two other PESs available in the literature near the global minimum of the PES. One can notice that the minimum reported by Tonolo et al.^4^ is given with respect to the ab initio data they have computed. The value of 1234.12 cm^–1^ does not correspond to the global minimum of the PES. In Table 4, the value of 1288 cm^–1^ refers to the global minimum obtained from their analytical PES. As can be seen, the interaction potentials reported by Tonolo et al.^4^ and Chahal et al.^5^ show deviations from our results, underestimating and overestimating our interaction energies by up to 30 and 20 cm^–1^, respectively. To gain insight into the discrepancies, we compare each PES with the corresponding ab initio calculations reported in each work, i.e., the CCSD­(T)-F12/AV­(Q + *d*)­Z for Tonolo et al.,^4^ the CCSD­(T)/CBS* ignoring the basis set superposition errors for Chahal et al.,^5^ and the CCSD­(T)-F12/AVTZ. We can see that our analytical PES closely reproduces the CCSD­(T)-F12/AVTZ ab initio, whereas the analytical representation of the PESs of Tonolo et al.^4^ and Chahal et al.^5^ fail to accurately capture the CCSD­(T)-F12/AV­(Q + *d*)­Z and CCSD­(T)/CBS* energy points, respectively. The deviations observed between the analytical PES of Tonolo et al.^4^ and the CCSD­(T)-F12/AV­(Q + *d*)­Z points are not surprising, as their PES was derived using only five H_2_ orientations and none of the five H_2_ orientations selected corresponded to a geometry providing the minimum energy of the PES. However, the significant discrepancies between the PES of Chahal et al.^5^ and the CCSD­(T)/CBS* energy points are much more surprising since Chahal et al.^5^ state that their fit is of spectroscopic accuracy with deviations less than 1 cm^–1^. The agreement between the analytical PES of Tonolo et al.,^4^ Chahal et al. is not part of the agreement, and the CCSD­(T)/CBS* is fortuitous, as both are approximations based on different levels of theory.

**Figure 4:**
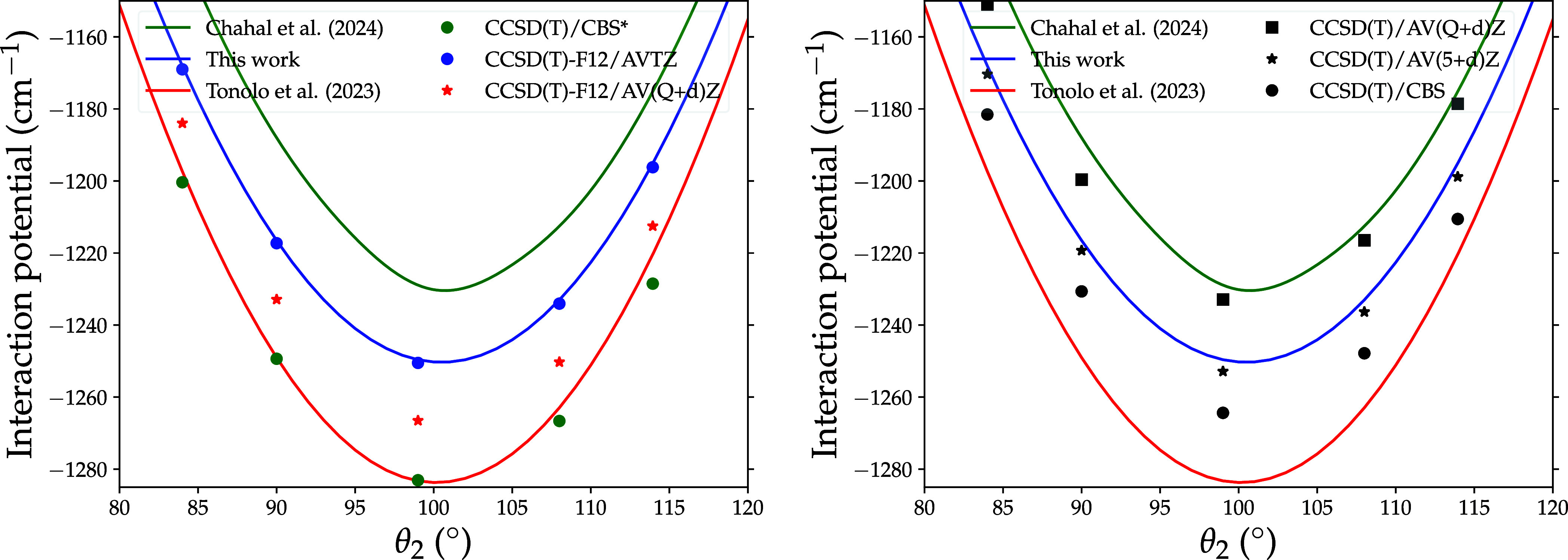
Comparison of the PO^+^-H_2_ interaction potential computed using different levels of theory and the analytical representations used in this work, in that of Tonolo et al.^4^ and in the work of Chahal et al.^5^ The CCSD­(T)-F12/AVTZ, CCSD­(T)-F12/AV­(Q + *d*)­Z, and CCSD­(T)/CBS* levels of theory were used in this work, that of Tonolo et al.^4^ and that of Chahal et al.,^5^ respectively. The calculations were performed for *R* = 5.25 *a*_0_, θ_1_ = 112° and ϕ = 0° which is near the equilibrium position.

To draw a robust conclusion, the right panel of Figure 4 assesses the accuracy of the levels of theory considered in these works, namely, CCSD­(T)-F12/AVTZ, CCSD­(T)-F12/AV­(Q + *d*)­Z, and CCSD­(T)/CBS* with respect to the ‘gold standard’ CCSD­(T) method in conjunction with the CBS limit derived by extrapolating the AV­(X + *d*)­Z (X = T, Q, 5) basis sets. It is important to note here that we mention two separate CBS extrapolation methods. Our analytical representation and that of Chahal et al.^5^ overestimate the CCSD­(T)/CBS interaction potential by up to 15 and 35 cm^–1^, respectively. The 15 cm^–1^ difference likely arises from the contribution of the *d*-functions, as the level of theory used in this work has the quality of the ‘gold standard’ CCSD­(T) method in conjunction with a complete basis set.^8^ In contrast, the analytical PES of Tonolo et al.^4^ underestimates the reference calculations by up to 20 cm^–1^. In the past, it has been shown that the use of the CCSD­(T)-F12 method together with large atomic basis sets could lead to an overestimation of the interaction energy. However, in the present case, the present overestimation could certainly be mainly attributed to the limited quality of the fit that has been performed on a limited number of ab initio energy points.

If one considers the CCSD­(T)/CBS interaction potential as the reference, the PES of Chahal et al.^5^ appears to be the least accurate. However, the differences in these three PESs (2–3%) as seen from the 1D cuts are relatively minor and cannot account for the large discrepancies (more than a factor of 2) observed between the collisional rate coefficients of Tonolo et al.^4^ and those of Chahal et al.^5^

### PO^+^-H_2_ Energy Levels

The rovibrational states of the complex PO^+^-H_2_ were obtained for the total angular momenta *J* = 0 to *J* = 3 using the methodologies described above. The calculation of the rovibrational states of a molecular system is a common way to probe the quality of the intermolecular interaction. For the PO^+^-H_2_ complex, there are no published experimental data to our knowledge. However, the quality of the electronic structure calculations carried out here and the experience derived from other similar van der Waals complexes suggest that the current results are a guide for future experimental investigations. In Tables 5, 6, 7, and 8, we present the lower rovibrational states of this system, reported relative to the ground rovibrational states of the PO^+^-*para-*H_2_ or PO^+^-*ortho*-H_2_ complexes, which are located at −830.6792 and −765.0750 cm^–1^, respectively, in the MCTDH calculations, and at −828.9783 and −761.3822 cm^–1^ using the BOUND package, relative to dissociation. The close agreement between the MCTDH and BOUND results, within approximately 2 cm^–1^, indicates that the MCTDH method provides a consistent and reliable description of the lower bound states of the system. Since BOUND is considered the reference, this close match validates the accuracy of the MCTDH calculations for these states.

**Table 5:** Low-Energy Rovibrational Levels of PO^+^-*para*-H_2_ and PO^+^-*ortho*-H_2_ for *J* = 0^a^

PO^+^-*para*-H_2_					PO^+^-*ortho*-H_2_				
assgt.	parity	MCTDH	wgt.	BOUND	assgt.	parity	MCTDH	wgt.	BOUND
Σ(0_00_)	+	0.00	0.2646	0.00	Σ(0_00_)	+	0.00	0.2432	0.00
Σ(0_00_)	+	120.4577	0.2289	120.3893	Σ(0_00_)	–	19.1412	0.2209	18.9134
Σ(0_00_)	+	213.8560	0.230	213.7158	Σ(0_00_)	+	122.4723	0.3315	122.4533
Σ(0_00_)	–	239.9980	0.2289	239.9738	Σ(0_00_)	–	139.0331	0.3236	138.8562

a For PO^+^-*para*-H_2_, the lowest energy is −830.6792 cm^–1^, and for PO^+^-*ortho*-H_2_, it is −765.0750 cm^–1^. ‘Wgt’ in the table is the weight of the dominant configuration (see text for more details). The units are given in cm^–1^.

**Table 6:** Same as Table 5 for *J* = 1

PO^+^-*para*-H_2_					PO^+^-*ortho*-H_2_				
assgt.	parity	MCTDH	Wgt.	BOUND	assgt.	parity	MCTDH	wgt.	BOUND
Σ(1_01_)	+	1.0982	0.2528	1.0367	Σ(1_01_)	+	1.0302	0.2717	0.9635
Σ(1_11_)	+	1.9711	0.3058	1.9059	Σ(1_11_)	+	1.8233	0.2843	1.7538
Σ(1_10_)	–	2.1730	0.3116	2.0549	Σ(1_10_)	–	1.9510	0.3082	1.8428
Σ(1_01_)	+	121.5322	0.3116	121.4197	Σ(1_11_)	–	20.2881	0.2433	20.0803
Σ(1_01_)	+	122.5592	0.2534	122.4473	Σ(1_01_)	–	21.2720	0.2433	21.0707
Σ(1_01_)	–	122.7591	0.2534	122.6133	Σ(1_10_)	+	21.5124	0.2433	21.3267
Σ(1_01_)	+	214.9682	0.2534	214.7458	Σ(1_11_)	+	123.4672	0.2433	123.4243
Σ(1_11_)	+	215.9422	0.3058	215.7697	Σ(1_11_)	+	124.3023	0.3372	124.2551
Σ(1_11_)	–	216.1514	0.2238	215.9483	Σ(0_00_)	–	124.4164	0.2927	124.3404
Σ(1_11_)	+	241.0442	0.2238	240.9168	Σ(1_01_)	–	140.1466	0.3372	139.9678
Σ(1_10_)	+	242.1163	0.2238	242.0034	Σ(1_01_)	–	141.4063	0.3082	141.2343
Σ(1_10_)	–	242.3061	0.3501	242.1085	Σ(1_10_)	+	141.6353	0.3082	141.4674

**Table 7:** Same as Table 5 for *J* = 2

PO^+^-*para*-H_2_			PO^+^-*ortho*-H_2_		
assgt.	MCTDH	wgt.	assgt.	MCTDH	Wgt.
Σ(2_02_)	3.2631	0.291	Σ(2_02_)	3.0781	0.3112
Σ(2_12_)	3.9661	0.273	Σ(2_12_)	3.7600	0.2433
Σ(2_11_)	4.5722	0.331	Σ(2_11_)	4.1484	0.2825
Π(2_21_)	6.9023	0.273	Σ(2_21_)	5.5322	0.3156
Π(2_20_)	6.9023	0.324	Σ(2_20_)	6.5491	0.2342
Σ(1_10_)	7.1841	0.202	Σ(1_01_)	22.5415	0.2633
Σ(1_11_)	7.2162	0.295	Σ(1_01_)	23.3206	0.3251
Σ(1_11_)	123.6443	0.294	Σ(1_10_)	24.0392	0.2743
Σ(1_01_)	124.4981	0.302	Σ(1_10_)	26.9665	0.3314
Σ(1_01_)	125.0962	0.313	Σ(1_01_)	27.0043	0.2842
Σ(1_11_)	128.1582	0.306	Σ(1_10_)	125.4491	0.3102
Σ(1_10_)	128.1854	0.342	Σ(0_10_)	126.1883	0.3101
Π(1_10_)	133.1732	0.342	Σ(1_11_)	126.5384	0.2831

**Table 8:** Same as Table 5 for *J* = 3

PO^+^-*para*-H_2_			PO^+^-*ortho*-H_2_		
assgt.	MCTDH	wgt.	assgt.	MCTDH	wgt.
Σ(3_03_)	6.4370	0.341	Σ(3_03_)	6.1182	0.232
Σ(3_13_)	6.9381	0.294	Σ(3_13_)	6.6562	0.312
Σ(3_12_)	8.1464	0.304	Σ(3_12_)	7.4423	0.286
Σ(3_22_)	10.4795	0.290	Σ(3_22_)	9.6506	0.316
Σ(3_21_)	10.6293	0.321	Σ(3_21_)	9.7352	0.298
Δ(3_31_)	13.6353	0.285	Σ(3_31_)	13.9762	0.308
Δ(3_30_)	13.6351	0.307	Σ(3_30_)	13.9784	0.323
Σ(3_22_)	15.3377	0.340	Σ(3_22_)	25.8303	0.304
Σ(3_21_)	15.3402	0.328	Σ(3_21_)	26.3725	0.301
Σ(1_10_)	126.7462	0.296	Σ(1_01_)	27.8036	0.292
Σ(1_01_)	127.3897	0.303	Σ(1_10_)	30.3831	0.302
Σ(1_11_)	128.5822	0.305	Σ(1_01_)	30.5673	0.307
Σ(1_10_)	131.3671	0.285	Π(1_10_)	35.6732	0.288
Σ(1_11_)	131.4950	0.311	Π(1_01_)	35.6733	0.303
Σ(1_10_)	136.9382	0.297	Σ(1_10_)	35.8123	0.303

The arrangement of low-energy levels reflects the anisotropy and depth of the potential energy surface, with a denser distribution near the bottom of the well. The overall structure and level positions are well reproduced, demonstrating good agreement between the two methods. Minor differences, likely due to distinct numerical treatments of the kinetic and potential energy operators, remain within acceptable limits and do not affect the overall consistency. This supports the robustness of the MCTDH approach in accurately describing bound states in weakly bound van der Waals systems.

The visualization of rovibrational states is done with an approach similar to what we did in our work on H_2_O-HCN.^31^ First, and as stated earlier, the *K*-Legendre DVR selected for the calculation helps in connecting the average value of the angular modes ϕ_1_ and ϕ_2_ to *m*_*A*_ and *m*_*B*_, respectively, and then to *K* = *m*_*A*_ + *m*_*B*_, with = *m*_*A*_ and *m*_*B*_ the projection of the rotational angular momentum of each monomer on the Space-Fixed *z*-axis *K* provides the major character (Σ, Π, Δ, and ···) of the computed eigenstates. Next, the rotational wave function of the H_2_ molecule can be described by |*j*_*B*_, *m*_*B*_⟩ with *j*_*B*_ as the total angular momentum and *m*_*B*_, the eigenvalue of *j*_*B*_ on the laboratory frame (Body-Fixed *Z*-axis).

The wave function can then write as follows

$$\left|\Psi \rangle =\sum_{j_{B}m_{B},\chi_{0}}C_{j_{B}m_{B},\chi_{0}}|j_{B},m_{B}\rangle |\chi_{0}\right\rangle$$ 9

where χ_0_ = {*j*_*B*_(*m*_*B*_);*n*_0_} with *j*_*A*_(*m*_*A*_) the quantum state of PO^+^ and *n*_0_ labels the radial basis functions. The coefficients of the wave function determine the contribution of each quantum state to the overall wave function and depend on the specific rovibrational state of the system. The expansion coefficients *C*_*j*_*B*_ *m*_*B*_,χ_0__ from eq 9 can be expanded as follows,

$$C_{j_{B}m_{B},\chi_{0}}=\overset{j_{B}}{\underset{-j_{B}}{\sum }}C_{j_{B}m_{B},\mu }\alpha^{\left(j_{B}m_{B}\right)}$$ 10

The coefficients α^(*j*_*B*_ *m*_*B*_)^ described in eq 10 can be obtained by diagonalizing the rotational Hamiltonian of the water monomer in the |*j*_*B*_, *m*_*B*_⟩ basis. We can then estimate the *para*-H_2_ and *ortho*-H_2_ rotational characters by projecting the wave function onto the rotational basis states of H_2_. The weights reported in the Tables 5–8 are the highest *p*_*i*_’s weight for a specific state and are obtained from *p*_*i*_ = ⟨Ψ|*P̂*_*i*_|Ψ⟩ where *P̂*_*i*_ = |*j*_*B*_, *m*_*B*_⟩⟨*j*_*B*_, *m*_*B*_|.

**Table 9:** Calculated Microwave Transition Frequencies (in cm^–1^) for PO^+^-*para*-H_2_

transition	MCTDH	BOUND	transition	MCTDH
1_11_ ← 0_00_	1.9711	1.9059	3_03_ ← 2_02_	3.1739
1_10_ ← 1_01_	1.0748	1.0182	3_13_ ← 2_12_	2.9720
2_02_ ← 1_01_	2.1648		3_12_ ← 2_11_	3.5742
2_12_ ← 1_11_	1.9950		3_12_ ← 3_03_	1.7094
2_11_ ← 1_10_	2.3992		3_22_ ← 2_21_	6.5134
2_12_ ← 1_01_	2.8679		3_21_ ← 2_20_	7.3662
2_11_ ← 2_02_	1.3091			

**Table 10:** Same as Table 9 for PO^+^-*ortho*-H_2_

transition	MCTDH	BOUND	transition	MCTDH
1_11_ ← 0_00_	1.8233	1.7538	3_03_ ← 2_02_	3.0401
1_10_ ← 1_01_	0.9208	0.8793	3_13_ ← 2_12_	2.8962
2_02_ ← 1_01_	2.0478		3_12_ ← 2_11_	3.2939
2_12_ ← 1_11_	1.9367		3_12_ ← 3_03_	1.3241
2_11_ ← 1_10_	2.1974		3_22_ ← 2_21_	4.1184
2_12_ ← 1_01_	2.7298		3_21_ ← 2_20_	3.1861
2_11_ ← 2_02_	1.0703			

The PO^+^-H_2_ system undergoes large-amplitude intermolecular vibrational motions. This raises questions about the average positions associated with the vibrational ground states. To quantify the deviation of the H_2_ moiety from the PO^+^ axis, we calculated the expected values ⟨θ_1_⟩ and ⟨θ_2_⟩, which represent the polar angles θ_1_ and θ_2_, respectively, for each vibrational state. These values are determined using ⟨θ_*i*_⟩ = cos^–1^⟨cosθ_*i*_⟩, where ⟨cosθ_*i*_⟩ is the expectation value of cosθ_*i*_ for *i* = 1, 2. For the ground state, the results indicate ⟨θ_1_⟩ = 111.8124° and ⟨θ_2_⟩ = 100.4728° for *para*-H_2_, and ⟨θ_1_⟩ = 111.7497° and ⟨θ_2_⟩ = 101.7328° for *ortho*-H_2_ as reported in Tables 13 and 14. These values suggest that the molecular axis of H_2_ is nearly perpendicular to the intermolecular axis of the PO^+^-H_2_ complex within approximately 20°. Due to the definition of the polar angles, which range from 0 to 180°, the expectation values ⟨θ_1_⟩ and ⟨θ_2_⟩ are always positive and nonzero, even when the equilibrium geometry is planar θ_1_ = θ_2_ = 0°. As in the case of NO^+^-H_2_,^50^ the vibrational states are classified into distinct modes: the in-plane shear swinging mode associated with the first vibrationally excited state (ν_1_), the PO^+^ - H_2_ symmetric stretching mode corresponding to the second vibrationally excited state (ν_2_), and the PO^+^ - H_2_ asymmetric stretching mode linked to the third vibrationally excited state (ν_3_). The corresponding modes are reported in Tables 11–14.

**Table 11:** Calculated Rotational Constants of PO^+^-*para*-H_2_ (in cm^–1^) for Each Vibrational State^a^

	MCTDH				BOUND			
state	energy	A	B	C	energy	A	B	C
ν_0_	–830.6792	1.5230	0.6500	0.4480	–828.9783	1.4615	0.5925	0.4435
ν_1_	–710.2215	1.6615	0.6345	0.4345	–708.5890	1.6260	0.5980	0.4320
ν_2_	–616.8232	1.6345	0.6605	0.4515	–615.2624	1.6285	0.6045	0.4255
ν_3_	–590.6812	1.690	0.6180	0.4280	–589.0045	1.6110	0.5240	0.4190

a The vibrational energies are given in units of cm^–1^.

The rovibrational state energies obtained from our calculations are then used to extract the frequency of the PO^+^-H_2_ rotational transition lines. Thirteen of these transition lines are presented in Tables 9 and 10. If we rely on studies on similar systems, such as CO-H_2_ ^51^ and HCN-H_2_ ^52^ (just to mention a couple of them), the theoretical data obtained for PO^+^-H_2_ are also expected to be of excellent quality. In fact, the results on similar van der Waals rigid-rotor systems have generally confirmed that such high-level ab initio calculations provide an accurate representation of intermolecular interactions and can be used confidently for spectroscopic analysis.

The transition frequencies are used to obtain the rotational constants, where, following the methodology outlined by Castro et al.,^53^ we have used the transition 1_01_ ← 0_00_, 1_11_ ← 0_00_ and 1_10_ ← 0_00_ to deduce the rotational constants *A*, *B* and *C* according to the formula: 1_01_ ← 0_00_ = *B* + *C*, 1_11_ ← 0_00_ = *A* + *C* and 1_10_ ← 0_00_ = *A* + *B*, with the added help of the near-prolate nature of the molecule which leads to an ordering of the rotational states as *A* > *B* > *C*. The rotational constants of PO^+^-H_2_ deduced from these calculations are presented in Tables 11 and 12. As shown by Orek et al.,^50^ the rotational constants could be determined for specific vibrational states, which is what we did and show in Tables 11 and 12 for selected vibrational states.

**Table 12:** Same as in Table 11 for PO^+^-*ortho*-H_2_

	MCTDH				BOUND			
state	energy	*A*	*B*	*C*	energy	*A*	*B*	*C*
ν_0_	–765.0759	1.3721	0.5792	0.4512	–761.382	1.3161	0.5261	0.4371
ν_1_	–745.9349	1.6775	0.6935	0.4535	–742.468	1.7020	0.7120	0.4550
ν_2_	–642.6039	1.3895	0.5545	0.4405	–638.928	1.3590	0.5280	0.4430
ν_3_	–626.0429	1.9310	0.6710	0.4420	–622.525	1.9385	0.6725	0.4395

The rotational constants exhibit notable correlations, particularly with increasing vibrational energy and the expectation value of the intermolecular distance, <*R*>. Specifically, as the vibrational energy increases, the rotational constants tend to decrease, reflecting the expansion of the molecular complex in excited vibrational states. This sensitivity underscores the strong coupling between vibrational and rotational motions. In the case of the PO^+^-H_2_ system, the combination of the small rotational constant of PO^+^ and the presence of a deep potential well allows for the existence of a large number of vibrational states for *J* = 0. A similar trend has been observed in other complexes of ion molecules, such as NO^+^-H_2_,^50^ further confirming the generality of this behavior in weakly bound molecular dimers containing a molecular ion.

The determination of the intermolecular vibrational states proved to be a particularly challenging task. As shown in Tables 13 and 14, these states were identified through a detailed analysis of contour plots that represent the wave functions of the corresponding eigenstates in carefully chosen internal coordinates. Additionally, wavepacket propagation provides an alternative approach for characterizing rovibrational states. In this work, this method^47^ was used by evaluating the average contributions of each degree of freedom.

**Table 13:** Characterization of the Calculated Vibrational States for PO^+^-pH_2_ ^a^

state	vibrational energy	<*R*>	Δ*R*	<θ_1_>	Δθ_1_	<θ_2_>	Δθ_2_
ν_0_	–830.6792	5.4504	0.3748	111.8124	6.8353	100.4728	14.1348
ν_1_	–710.2215	5.6321	0.4453	114.5085	12.1493	100.6235	14.3610
ν_2_	–616.8232	5.7761	0.5716	113.6633	14.4697	100.3649	14.8305
ν_3_	–590.6812	5.8244	0.5960	115.1591	12.2126	100.3123	14.8829

a The vibrational energies are given in cm^–1^; the parameters <*R*> and Δ*R* are given in bohr; <θ_1_>, Δθ_1_, <θ_2_>, and Δθ_2_ are in degrees and are defined in the text.

Our methodology for characterizing vibrational states remained consistent with previously established approaches, particularly those successfully applied in the identification of vibrational states for H_2_O–HCN^31^ and H_2_O–H_2_.^47^

The characterization of intermolecular vibrational states is crucial to understanding the quantum dynamics of weakly bound molecular complexes. However, excitation energies alone are often insufficient; they must be assigned to fundamental vibrations. This requires access to wave functions, which are obtained through relaxation procedures. During relaxation, the initial wave packet evolves and converges toward the stationary bound states, with each resulting eigenstate saved as a separate file in the output representing the corresponding wave function. The analysis of wave functions provides insight into the nature of bound states and the interplay between rotational and vibrational motions.

To explore these aspects, we present the wave functions for each system configuration in Figures 5 and 6. Additional insights are obtained by examining two-dimensional contour plots of the wave function in carefully selected coordinate pairs. For each state, we analyze 2D contour plots as functions of (*R*, θ_1_), with specific visualizations for PO^+^-*para*-H_2_ and PO^+^-*ortho*-H_2_ provided in Figures 5a and 6a. In Tables 13 and 14 the quantities Δ*R* and Δθ_*i*_ reported correspond to the root-mean-square (rms) amplitudes of the coordinate fluctuations. These values are used to describe the spatial extent of vibrational motion and the variation of the coordinates from one state to another. In the ground state, the wave function is localized at the global minimum, with H_2_ moving around PO^+^ in a manner consistent with localization at <*R*≥ 5.450 bohr and <θ_1_≥ 111.812°. Figures 5b,c and 6b,c show the contour plots of the wave function of the first excited states for PO^+^-*para*-H_2_ and PO^+^-*ortho*-H_2_. In particular, there is a distinct change in the nature of the complex between the ground and first vibrational states for *J* = 0, indicating a sudden change in the geometry between these states. Figure 5c,d show the wave functions of the second and third excited states of *para*-H_2_ with two nodes with respect to the intermolecular distance *R* and θ_1_. In Figure 6, the same characterization has been done, as shown in Figure 6a–d, for *ortho*-H_2_, simplifying the process of recognizing the intermolecular stretch and its various excitations as reported in Table 14. In the *ortho*-H_2_ configuration, the ground state and the first excited state appear to be visually identical, showing a stable wave function without a node. Likewise, the second and third excited states exhibit a single node following R and θ_1_. However, the task of characterization of states primarily associated with excitations of the angular degrees of freedom in the complex proves to be considerably more demanding. Unfortunately, there is a lack of spectroscopic data in the existing literature on excited intermolecular vibrational states of the PO^+^-H_2_ complex, precluding any comparison with theoretical results.

**Table 14:** Same as Table 13 for PO^+^-oH_2_

state	vibrational energy	<*R*>	Δ*R*	<θ_1_>	Δθ_1_	<θ_2_>	Δθ_2_
ν_0_	–765.0759	5.4524	0.3716	111.7497	6.7723	101.7328	12.5415
ν_1_	–745.934	5.4705	0.3742	111.8342	6.8583	101.7340	12.7338
ν_2_	–642.6039	5.6095	0.4390	114.3594	11.9117	101.5396	13.5519
ν_3_	–626.0429	5.6302	0.4446	114.5564	12.0836	101.4435	12.860

**Figure 5:**
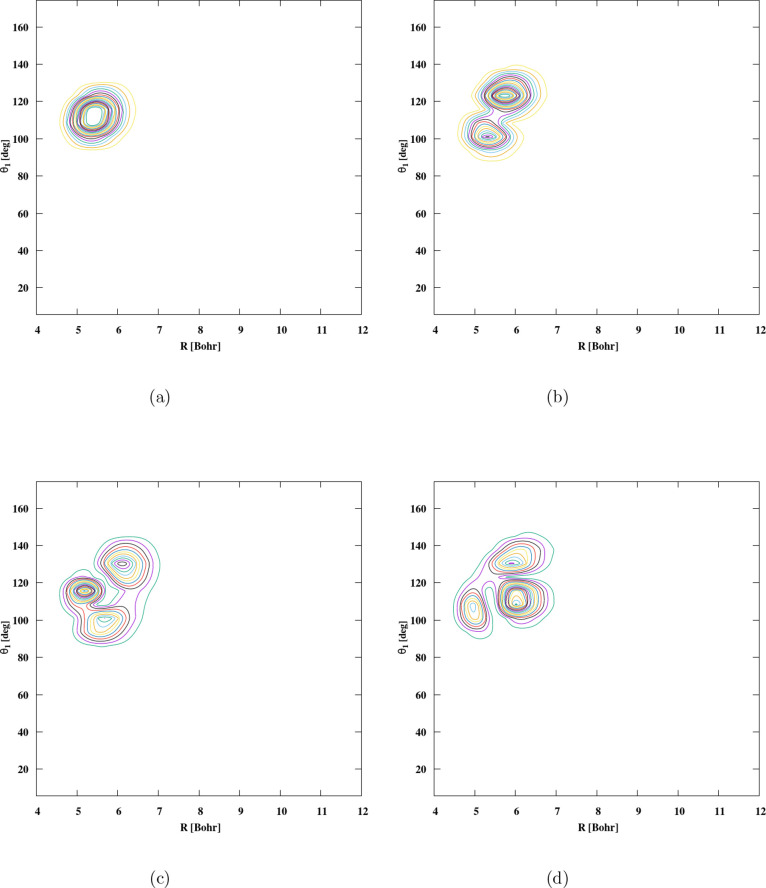
2D cuts of the wave function’s density of the lowest 4 states of PO^+^-*para*-H_2_. (a) *E* = 0 cm^–1^. (b) *E* = 120.4577 cm^–1^. (c) *E* = 213.8560 cm^–1^. (d) *E* = 239.9980 cm^–1^.

**Figure 6:**
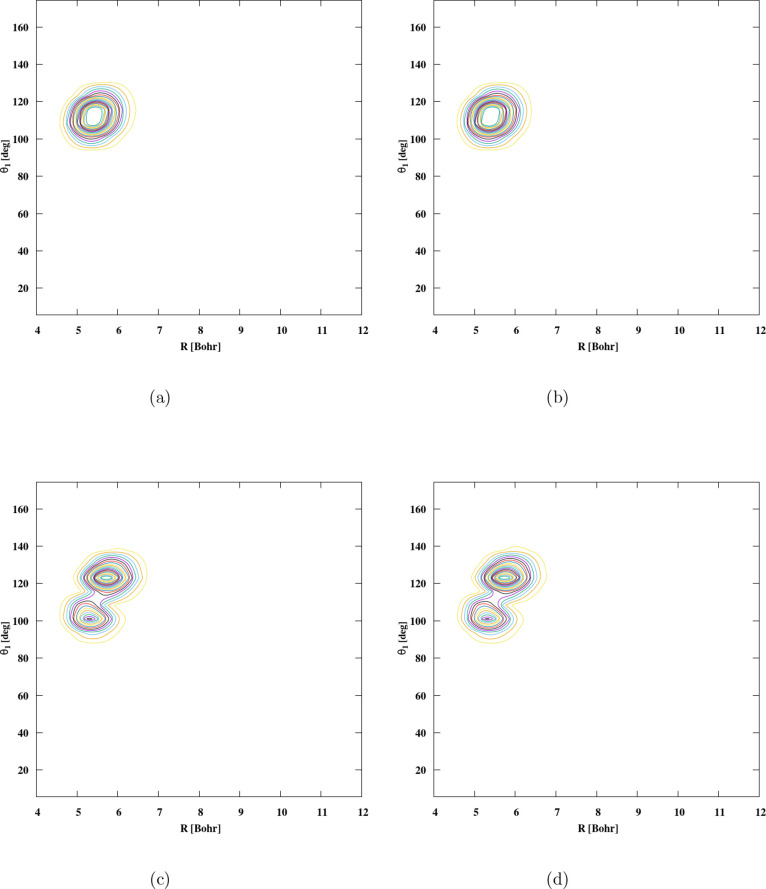
2D cuts of the density (*R*, θ_1_) of the wave functions of PO^+^-*ortho*-H_2_: (a) *E* = 0 cm^–1^, (b) *E* = 19.1412 cm^–1^, (c) *E* = 122.4723 cm^–1^, and (d) *E* = 139.0331 cm^–1^.

## Conclusions

We presented a new intermolecular PES for the PO^+^-H_2_ van der Waals system, calculated at the CCSD­(T)-F12 level of theory. Using the MCTDH method, we computed and analyzed the low-lying rovibrational levels of this system for values of the total angular momentum quantum number *J* between 0 and 3. Using an approach presented before,^30,31^ it is straightforward to assign the rovibrational states of the PO^+^-H_2_ complex based on the value of *K*, the projection of the total angular momentum *J*. To the best of our knowledge, this is the first time that the MCTDH approach has been used for rovibrational calculations on this type of van der Waals complex.

Because of the small rotational constant of PO^+^, a large rotational basis is required to converge the rovibrational states. When we add this to the dimensionality of the problem, the calculations for this type of system are challenging to converge with standard computation methods, such as the one implemented in BOUND, and would therefore benefit from the better scalability of algorithms such as MCTDH. As such, one of the objectives of this work was to offer the MCTDH method described here as a possible alternative for the systematic study of similar systems. Our results could also be expected to guide future experimental work on this complex.

Finally, as mentioned in the Introduction, the main purpose of this work was to build a new PES that could later be used to settle the debate that arises from the large differences observed in cross sections and collision rate coefficients obtained by Tonolo et al.^4^ and Chahal et al.^5^ To validate the accuracy of the PES, we performed parallel calculations using both the MCTDH and BOUND packages. The zero-point energies obtained with MCTDH were 422.201 cm^–1^ for PO^+^- *para*-H_2_ and 487.805 cm^–1^ for PO^+^-*ortho*-H_2_, while the BOUND package produced 423.901 and 491.497 cm^–1^, respectively. The close agreement between the two methods shows that both MCTDH and bound calculations converge. For future work, we will employ both approaches, with particular emphasis on MCTDH, which is well-suited for treating systems with a high density of states and delivering benchmark-quality results.
